# Early short course of neuromuscular blocking agents in patients with COVID-19 ARDS: a propensity score analysis

**DOI:** 10.1186/s13054-022-03983-5

**Published:** 2022-05-17

**Authors:** Gianluigi Li Bassi, Kristen Gibbons, Jacky Y. Suen, Heidi J. Dalton, Nicole White, Amanda Corley, Sally Shrapnel, Samuel Hinton, Simon Forsyth, John G. Laffey, Eddy Fan, Jonathon P. Fanning, Mauro Panigada, Robert Bartlett, Daniel Brodie, Aidan Burrell, Davide Chiumello, Alyaa Elhazmi, Mariano Esperatti, Giacomo Grasselli, Carol Hodgson, Shingo Ichiba, Carlos Luna, Eva Marwali, Laura Merson, Srinivas Murthy, Alistair Nichol, Mark Ogino, Paolo Pelosi, Antoni Torres, Pauline Yeung Ng, John F. Fraser, The COVID-19 Critical Care Consortium, Tala Al-Dabbous, Huda Alfoudri, Mohammed Shamsah, Subbarao Elapavaluru, Ashley Berg, Christina Horn, Yunis Mayasi, Stephan Schroll, Dan Meyer, Jorge Velazco, Ludmyla Ploskanych, Wanda Fikes, Rohini Bagewadi, Marvin Dao, Haley White, Dan Meyer, Ashley Ehlers, Maysoon Shalabi-McGuire, Trent Witt, Lorenzo Grazioli, Luca Lorini, E. Wilson Grandin, Jose Nunez, Tiago Reyes, Diarmuid OBriain, Stephanie Hunter, Mahesh Ramanan, Julia Affleck, Hemanth Hurkadli Veerendra, Sumeet Rai, Josie Russell-Brown, Mary Nourse, Mark Joseph, Brook Mitchell, Martha Tenzer, Ryuzo Abe, Hwa Jin Cho, In Seok Jeong, Nadeem Rahman, Vivek Kakar, Nicolas Brozzi, Omar Mehkri, Sudhir Krishnan, Abhijit Duggal, Stuart Houltham, Jerónimo Graf, Roderigo Diaz, Roderigo Orrego, Camila Delgado, Joyce González, Maria Soledad Sanchez, Michael Piagnerelli, Josefa Valenzuela Sarrazin, A./Prof. Gustavo Zabert, Lucio Espinosa, Paulo Delgado, Victoria Delgado, Diego Fernando Bautista Rincón, Angela Maria Marulanda Yanten, Melissa Bustamante Duque, Daniel Brodie, Alyaa Elhazmi, Abdullah Al-Hudaib, Maria Callahan, M. Azhari Taufik, Elizabeth Yasmin Wardoyo, Margaretha Gunawan, Nurindah S. Trisnaningrum, Vera Irawany, Muhammad Rayhan, Mauro Panigada, Antonia Pesenti, Alberto Zanella, Michela Leone, Giacomo Grasselli, Silvia Coppola, Sebastiano Colombo, Massimo Antonelli, Simone Carelli, Domenico L. Grieco, Motohiro Asaki, Kota Hoshino, Leonardo Salazar, Laura Duarte, John Laffey, Bairbre McNicholas, David Cosgrave, Joseph McCaffrey, Allison Bone, Yusuff Hakeem, James Winearls, Mandy Tallott, David Thomson, Christel Arnold-Day, Jerome Cupido, Zainap Fanie, Malcom Miller, Lisa Seymore, Dawid van Straaten, Ali Ait Hssain, Jeffrey Aliudin, Al-Reem Alqahtani, Khoulod Mohamed, Ahmed Mohamed, Darwin Tan, Joy Villanueva, Ahmed Zaqout, Ethan Kurtzman, Arben Ademi, Ana Dobrita, Khadija El Aoudi, Juliet Segura, Gezy Giwangkancana, Shinichiro Ohshimo, Koji Hoshino, Saito Hitoshi, Javier Osatnik, Anne Joosten, Antoni Torres, Minlan Yang, Ana Motos, Carlos Luna, Francisco Arancibia, Virginie Williams, Alexandre Noel, Nestor Luque, Trieu Huynh Trung, Sophie Yacoub, Marina Fantini, Ruth Noemi Jorge García, Enrique Chicote Alvarez, Anna Greti, Adrian Ceccato, Angel Sanchez, Ana Loza Vazquez, Ferran Roche-Campo, Diego Franch-Llasat, Divina Tuazon, Marcelo Amato, Luciana Cassimiro, Flavio Pola, Francis Ribeiro, Guilherme Fonseca, Heidi Dalton, Mehul Desai, Erik Osborn, Hala Deeb, Antonio Arcadipane, Gennaro Martucci, Giovanna Panarello, Chiara Vitiello, Claudia Bianco, Giovanna Occhipinti, Matteo Rossetti, Raffaele Cuffaro, Sung-Min Cho, Hiroaki Shimizu, Naoki Moriyama, Jae-Burm Kim, Nobuya Kitamura, Johannes Gebauer, Toshiki Yokoyama, Abdulrahman Al-Fares, Sarah Buabbas, Esam Alamad, Fatma Alawadhi, Kalthoum Alawadi, Hiro Tanaka, Satoru Hashimoto, Masaki Yamazaki, Tak-Hyuck Oh, Mark Epler, Cathleen Forney, Louise Kruse, Jared Feister, Joelle Williamson, Katherine Grobengieser, Eric Gnall, Sasha Golden, Mara Caroline, Timothy Shapiro, Colleen Karaj, Lisa Thome, Lynn Sher, Mark Vanderland, Mary Welch, Sherry McDermott, Matthew Brain, Sarah Mineall, Dai Kimura, Luca Brazzi, Gabriele Sales, Tawnya Ogston, Dave Nagpal, Karlee Fischer, Roberto Lorusso, Rajavardhan Rangappa, Sujin Rai, Argin Appu, Mariano Esperatti, Diarmuid OBriain, Edmund G. Carton, Ayan Sen, Amanda Palacios, Deborah Rainey, Gordan Samoukoviv, Josie Campisi, Lucia Durham, Emily Neumann, Cassandra Seefeldt, Octavio Falcucci, Amanda Emmrich, Jennifer Guy, Carling Johns, Kelly Potzner, Catherine Zimmermann, Angelia Espinal, Nina Buchtele, Michael Schwameis, Stephanie-Susanne Stecher, Delila Singh, Michaela Barnikel, Lukas Arenz, Akram Zaaqoq, Lan Anh Galloway, Caitlin Merley, Alistair Nichol, Marc Csete, Luisa Quesada, Isabela Saba, Daisuke Kasugai, Hiroaki Hiraiwa, Taku Tanaka, Eva Marwali, Yoel Purnama, Santi Rahayu Dewayanti, Ardiyan, Dafsah Arifa Juzar, Debby Siagian, Yih-Sharng Chen, Mark Ogino, Indrek Ratsep, Getter Oigus, Kristo Erikson, Andra-Maris Post, Lauri Enneveer, Piret Sillaots, Frank Manetta, Effe Mihelis, Iam Claire Sarmiento, Mangala Narasimhan, Michael Varrone, Mamoru Komats, Julia Garcia-Diaz, Catherine Harmon, S. Veena Satyapriya, Amar Bhatt, Nahush A. Mokadam, Alberto Uribe, Alicia Gonzalez, Haixia Shi, Johnny McKeown, Joshua Pasek, Juan Fiorda, Marco Echeverria, Rita Moreno, Bishoy Zakhary, Marco Cavana, Alberto Cucino, Giuseppe Foti, Marco Giani, Vincenzo Russotto, Davide Chiumello, Valentina Castagna, Andrea DellAmore, Paolo Navalesi, Hoi-Ping Shum, Alain Vuysteke, Asad Usman, Andrew Acker, Benjamin Smood, Blake Mergler, Federico Sertic, Madhu Subramanian, Alexandra Sperry, Nicolas Rizer, Erlina Burhan, Menaldi Rasmin, Ernita Akmal, Faya Sitompul, Navy Lolong, Bhat Naivedh, Simon Erickson, Peter Barrett, David Dean, Julia Daugherty, Antonio Loforte, Irfan Khan, Mohammed Abraar Quraishi, Olivia DeSantis, Dominic So, Darshana Kandamby, Jose M. Mandei, Hans Natanael, Eka YudhaLantang, Anastasia Lantang, Surya Oto Wijaya, Anna Jung, George Ng, Wing Yiu Ng, Pauline Yeung Ng, Shu Fang, Alexis Tabah, Megan Ratcliffe, Maree Duroux, Shingo Adachi, Shota Nakao, Pablo Blanco, Ana Prieto, Jesús Sánchez, Meghan Nicholson, Warwick Butt, Alyssa Serratore, Carmel Delzoppo, Pierre Janin, Elizabeth Yarad, Richard Totaro, Jennifer Coles, Bambang Pujo, Robert Balk, Andy Vissing, Esha Kapania, James Hays, Samuel Fox, Garrett Yantosh, Pavel Mishin, Saptadi Yuliarto, Kohar Hari Santoso, Susanthy Djajalaksana, Arie Zainul Fatoni, Masahiro Fukuda, Keibun Liu, Paolo Pelosi, Denise Battaglini, Juan Fernando Masa Jiménez, Diego Bastos, Sérgio Gaião, Desy Rusmawatiningtyas, Jessica Buchner, Young-Jae Cho, Su Hwan Lee, Tatsuya Kawasaki, Laveena Munshi, Pranya Sakiyalak, Prompak Nitayavardhana, Tamara Seitz, Rakesh Arora, David Kent, Daniel Marino, Swapnil Parwar, Andrew Cheng, Jennene Miller, Shigeki Fujitani, Naoki Shimizu, Jai Madhok, Clark Owyang, Hergen Buscher, Claire Reynolds, Olavi Maasikas, Aleksan Beljantsev, Vladislav Mihnovits, Takako Akimoto, Mariko Aizawa, Kanako Horibe, Ryota Onodera, Carol Hodgson, Aidan Burrell, Meredith Young, Timothy George, Kiran Shekar, Niki McGuinness, Lacey Irvine, Brigid Flynn, Tomoyuki Endo, Kazuhiro Sugiyama, Keiki Shimizu, Eddy Fan, Kathleen Exconde, Shingo Ichiba, Leslie Lussier, Gösta Lotz, Maximilian Malfertheiner, Lars Maier, Esther Dreier, Neurinda Permata Kusumastuti, Colin McCloskey, Al-Awwab Dabaliz, Tarek B. Elshazly, Josiah Smith, Konstanty S. Szuldrzynski, Piotr Bielański, Yusuff Hakeem, Keith Wille, Srinivas Murthy, Ken Kuljit S. Parhar, Kirsten M. Fiest, Cassidy Codan, Anmol Shahid, Mohamed Fayed, Timothy Evans, Rebekah Garcia, Ashley Gutierrez, Hiroaki Shimizu, Tae Song, Rebecca Rose, Suzanne Bennett, Denise Richardson, Giles Peek, Lovkesh Arora, Kristina Rappapport, Kristina Rudolph, Zita Sibenaller, Lori Stout, Alicia Walter, Daniel Herr, Nazli Vedadi, Robert Bartlett, Antonio Pesenti, Shaun Thompson, Lace Sindt, Sean Rajnic, Cale Ewald, Julie Hoffman, Xiaonan Ying, Ryan Kennedy, Matthew Griffee, Anna Ciullo, Yuri Kida, Ricard Ferrer Roca, JordI Riera, Sofia Contreras, Cynthia Alegre, Christy Kay, Irene Fischer, Elizabeth Renner, Hayato Taniguci, John Fraser, Gianluigi Li Bassi, Jacky Suen, Adrian Barnett, Nicole White, Kristen Gibbons, Simon Forsyth, Amanda Corley, India Pearse, Samuel Hinton, Gabriella Abbate, Halah Hassan, Silver Heinsar, Varun A. Karnik, Katrina Ki, Hollier F. ONeill, Nchafatso Obonyo, Leticia Pretti Pimenta, Janice D. Reid, Kei Sato, Kiran Shekar, Aapeli Vuorinen, Karin S. Wildi, Emily S. Wood, Stephanie Yerkovich, James Lee, Daniel Plotkin, Barbara Wanjiru Citarella, Laura Merson, Emma Hartley, Bastian Lubis, Takanari Ikeyama, Balu Bhaskar, Jae-Seung Jung, Shay McGuinness, Glenn Eastwood, Sandra Rossi Marta, Fabio Guarracino, Stacy Gerle, Emily Coxon, Bruno Claro, Daniel Loverde, Namrata Patil, Vieri Parrini, Angela McBride, Kathryn Negaard, Angela Ratsch, Ahmad Abdelaziz, Juan David Uribe, Adriano Peris, Mark Sanders, Dominic Emerson, Muhammad Kamal, Pedro Povoa, Roland Francis, Ali Cherif, Sunimol Joseph, Matteo Di Nardo, Micheal Heard, Kimberly Kyle, Ray A. Blackwell, Michael Piagnerelli, Patrick Biston, Hye Won Jeong, Reanna Smith, Yogi Prawira, Giorgia Montrucchio, Arturo Huerta Garcia, Nahikari Salterain, Bart Meyns, Marsha Moreno, Rajat Walia, Amit Mehta, Annette Schweda, Moh Supriatna, Cenk Kirakli, Melissa Williams, Kyung Hoon Kim, Alexandra Assad, Estefania Giraldo, Wojtek Karolak, Martin Balik, Elizabeth Pocock, Evan Gajkowski, Kanamoto Masafumi, Nicholas Barrett, Yoshihiro Takeyama, Sunghoon Park, Faizan Amin, Fina Meilyana Andriyani, Serhii Sudakevych, Angela Ratsch, Magdalena Vera, Rodrigo Cornejo, Patrícia Schwarz, Ana Carolina Mardini, Thais de Paula, Ary Serpa Neto, Andrea Villoldo, Alexandre Siciliano Colafranceschi, Alejandro Ubeda Iglesias, Juan Granjean, Lívia Maria Garcia Melro, Giovana Fioravante Romualdo, Diego Gaia, Helmgton Souza, Filomena Galas, Rafael Máñez Mendiluce, Alejandra Sosa, Ignacio Martinez, Hiroshi Kurosawa, Juan Salgado, Beate Eric Hugi-MayrCharbonneau, Vitor Salvatore Barzilai, Veronica Monteiro, Rodrigo Ribeiro de Souza, Michael Harper, Hiroyuki Suzuki, Celina Adams, Jorge Brieva, George Nyale, Faisal Saleem Eltatar, Jihan Fatani, Husam Baeissa, Ayman AL Masri, Ahmed Rabie, Mok Yee Hui, Masahiro Yamane, Hanna Jung, Ayorinde Mojisola Margaret, Newell Nacpil, Katja Ruck, Rhonda Bakken, Claire Jara, Tim Felton, Lorenzo Berra, Bobby Shah, Arpan Chakraborty, Monika Cardona, Gerry Capatos, Bindu Akkanti, Abiodun Orija, Harsh Jain, Asami Ito, Brahim Housni, Sennen Low, Koji Iihara, Joselito Chavez, Kollengode Ramanathan, Gustavo Zabert, Krubin Naidoo, Ian Seppelt, Marlice VanDyk, Sarah MacDonald, Shingo Ichiba, Randy McGregor, Teka Siebenaler, Hannah Flynn, Kristi Lofton, Toshiyuki Aokage, Kazuaki Shigemitsu, Andrea Moscatelli, Giuseppe Fiorentino, Matthias Baumgaertel, Serge Eddy Mba, Jana Assy, Amelya Hutahaean, Holly Roush, Kay A. Sichting, Francesco Alessandri, Debra Burns, Ahmed Rabie, Gavin Salt, Carl P. Garabedian, Jonathan Millar, Malcolm Sim, Adrian Mattke, Danny McAuley, Jawad Tadili, Tim Frenzel, Yaron Bar-Lavie, Aaron Blandino Ortiz, Jackie Stone, Alexis Tabah, Antony Attokaran, Michael Farquharson, Brij Patel, Derek Gunning, Kenneth Baillie, Pia Watson, Kenji Tamai, Gede Ketut Sajinadiyasa, Dyah Kanyawati, Marcello Salgado, Assad Sassine, Bhirowo Yudo, Scott McCaul, Bongjin Lee, Sang Min Lee, Arnon Afek, Yoshiaki Iwashita, Bambang Pujo Semedi, Neurinda Permata Kusumastuti, Jack Metiva, Nicole Van Belle, Ignacio Martin-Loeches, Lenny Ivatt, Chia Yew Woon, Hyun Mi Kang, Timothy Smith, Erskine James, Nawar Al-Rawas, Yudai Iwasaki, Kenny Chan King-Chung, Vadim Gudzenko, Beate Hugi-Mayr, Fabio Taccone, Fajar Perdhana, Yoan Lamarche, Joao Miguel Ribeiro, Nikola Bradic, Klaartje Van den Bossche, Oude Lansink, Gurmeet Singh, Gerdy Debeuckelaere, Henry T. Stelfox, Cassia Yi, Jennifer Elia, Thomas Tribble, Shyam Shankar, Raj Padmanabhan, Bill Hallinan, Luca Paoletti, Yolanda Leyva, Tatuma Fykuda, Jenelle Badulak, Jillian Koch, Amy Hackman, Lisa Janowaik, Deb Hernandez, Jennifer Osofsky, Katia Donadello, Aizah Lawang, Josh Fine, Benjamin Davidson, Andres Oswaldo Razo Vazquez

**Affiliations:** 1 Critical Care Research Group, The Prince Charles Hospital, 627 Rode Rd, Chermside, Brisbane, QLD 4032 Australia; 2 University of Queensland, Brisbane, Australia; 3 Institut dInvestigacions Biomèdiques August Pi i Sunyer, Barcelona, Spain; 4 Queensland University of Technology, Brisbane, Australia; 5 UnitingCare Hospitals, Brisbane, Australia; 6 Wesley Medical Research, Brisbane, Australia; 7 Child Health Research Centre, The University of Queensland, Brisbane, QLD Australia; 8 INOVA Fairfax Medical Center, Heart and Vascular Institute, Falls Church, VA USA; 9 The Australian Research Council Centre of Excellence for Engineered Quantum Systems (EQUS, CE170100009), Brisbane, Australia; 10 Anaesthesia and Intensive Care Medicine, School of Medicine, National University of Ireland, and Galway University Hospitals, Galway, Ireland; 11 Interdepartmental Division of Critical Care Medicine, University of Toronto, Toronto, Canada; 12 Fondazione IRCCS Ca Granda Ospedale Maggiore Policlinico di Milano, Milan, Italy; 13 University of Michigan Medical Center, Ann Arbor, MI USA; 14 Department of Medicine, Columbia College of Physicians and Surgeons, and Center for Acute Respiratory Failure, New-York-Presbyterian Hospital, New York, NY USA; 15 Australian and New Zealand Intensive Care Research Centre, School of Public Health, Monash University, Melbourne, Australia; 16 Ospedale San Paolo, Milan, Italy; 17 University of Milan, Milan, Italy; 18 King Faisal Specialist Hospital and Research Centre, Riyadh, Saudi Arabia; 19 Hospital Privado de Comunidad, Escuela de Medicina, Universidad Nacional de Mar del Plata, Mar del Plata, Argentina; 20 Nippon Medical School Hospital, Tokyo, Japan; 21 Neumonología, Hospital de Clínicas, UBA, Buenos Aires, Argentina; 22 National Cardiovascular Center Harapan Kita, Jakarta, Indonesia; 23 ISARIC, Centre for Tropical Medicine and Global Health, University of Oxford, Oxford, UK; 24 Department of Pediatrics, Faculty of Medicine, University of British Columbia, Vancouver, Canada; 25 BC Childrens Hospital Research Institute, Vancouver, Canada; 26 University College Dublin-Clinical Research Centre at St Vincents University Hospital, Dublin, Ireland; 27 Department of Intensive Care, The Alfred Hospital, Melbourne, Australia; 28 Nemours Alfred I duPont Hospital for Children, Wilmington, DE USA; 29 Department of Surgical Sciences and Integrated Diagnostics, University of Genoa, Genoa, Italy; 30 Anesthesia and Critical Care, San Martino Policlinico Hospital, IRCCS for Oncology and Neurosciences, Genoa, Italy; 31 Hospital Clinic of Barcelona, Barcelona, Spain; 32 Department of Medicine, The University of Hong Kong, Pok Fu Lam, Hong Kong; 33 Al Adan Hospital, Hadiya, Kuwait; 34 Allegheny General Hospital, Pittsburgh, USA; 35 Avera McKennan Hospital & University Health Centre, Sioux Falls, USA; 36 Barmherzige Bruder Regansburg, Regensburg, Germany; 37 Baylor Scott & White Health, Dallas, USA; 38 Baylor University Medical Centre, Dallas, USA; 39 Bergamo Hospital, Bergamo, Italy; 40 Beth Israel Deaconess Medical Centre, Boston, USA; 41 Box Hill Hospital, Box Hill, Australia; 42 Caboolture Hospital, Caboolture, Australia; 43 Canberra Hospital, Canberra, Australia; 44 Carilion Clinic, Roanoke, USA; 45 Chiba University Graduate School of Medicine, Chiba, Japan; 46 Chonnam National University Hospital, Gwangju, South Korea; 47 Cleveland Clinic - Abu Dhabi, Abu Dhabi, UAE; 48 Cleveland Clinic - Florida, Weston, USA; 49 Cleveland Clinic - Ohio, Cleveland, USA; 50 Clinica Alemana de Santiago, Santiago, Chile; 51 Clinica Las Condez, Las Condes, Chile; 52 Clinica Pasteur National- University of Comahue, Neuquén, Argentina; 53 Linica Valle de Lilli, Cali, Colombia; 54 Medical ICU, Columbia College of Physicians and Surgeons, New-York-Presbyterian Hospital, New York, NY USA; 55 Dr Sulaiman Alhabib Medical Group – Research Center, Riyadh, Saudi Arabia; 56 Emory University Healthcare System, Atlanta, USA; 57 Fatmawati Hospital, Fatmawati, Indonesia; 58 Fondazione IRCCS Policlinico of Milan (Fondazione IRCCS Ca Granda Ospedale Maggiore Policlinico), Milan, Italy; 59 Fondazione Policlinico Universitario Agostino Gemelli IRCCS, Rome, Italy; 60 Fujieda Municipal General Hospital, Fujieda, Japan; 61 Fukuoka University, Fukuoka, Japan; 62 Fundación Cardiovascular de Colombia, Bogota, Colombia; 63 Galway University Hospitals, Galway, Ireland; 64 Geelong Hospital, Geelong, Australia; 65 Glenfield Hospital, Leicester, UK; 66 Gold Coast University Hospital, Gold Coast, Australia; 67 Groote Schuur Hospital, Cape Town, South Africa; 68 Hamad General Hospital - Weill Cornell Medical College in Qatar, Ar-Rayyan, Qatar; 69 Hartford HealthCare, Hartford, USA; 70 Hasan Sadikin Hospital (Adult), Jawa Barat, Indonesia; 71 Hiroshima University, Hiroshima, Japan; 72 Hokkaido University Hospital, Sapporo, Japan; 73 Hospital Alemán, Buenos Aires, Argentina; 74 Hospital Civil Marie Curie, Charleroi, Belgium; 75 Hospital Clinic, Barcelona, Spain; 76 Hospital de Clínicas, Buenos Aires, Argentina; 77 Hospital del Tórax, Barcelona, Spain; 78 Hospital du Sacre Coeur (Universite de Montreal), Montréal, Canada; 79 Hospital Emergencia Ate Vitarte, Ate, Peru; 80 Hospital for Tropical Diseases, London, UK; 81 Hospital Mater Dei, Msida, Malta; 82 Hospital Nuestra Señora de Gracia, Zaragoza, Spain; 83 Hospital Puerta de Hierro, Zapopan, Mexico; 84 Hospital Universitari Sagrat Cor, Barcelona, Spain; 85 Hospital Universitario Sant Joan dAlacant, Alicante, Spain; 86 Hospital Universitario Virgen de Valme, Sevilla, Spain; 87 Hospital Verge de La Cinta de Tortosa, Tortosa, Spain; 88 Houston Methodist Hospital, Houston, USA; 89 INCOR (Universidade de São Paulo), São Paulo, Brazil; 90 INOVA Fairfax Hospital, Falls Church, USA; 91 ISMETT, Palermo, Italy; 92 Johns Hopkins, Baltimore, USA; 93 Kakogawa Acute Care Medical Center, Kakogawa, Japan; 94 Keimyung University Dong San Hospital, Dalseo-gu, South Korea; 95 Kimitsu Chuo Hospital, Chiba, Japan; 96 Klinikum Passau, Passau, Germany; 97 Kouritu Tousei Hospital, Kaga, Japan; 98 Al-Amiri and Jaber Al-Ahmed Hospitals, Kuwait Extracorporeal Life Support Program, Kuwait City, Kuwait; 99 Kyoto Medical Centre, Kyoto, Japan; 100 Kyoto Prefectural University of Medicine, Kyoto, Japan; 101 Kyung Pook National University Chilgok Hospital, Daegu City, South Korea; 102 Lancaster General Health, Lancaster, USA; 103 Lankenau Institute of Medical Research (Main Line Health), Wynnewood, USA; 104 Launceston General Hospital, Launceston, Australia; 105 Le Bonheur Childrens Hospital, Memphis, USA; 106 Le Molinette Hospital (Ospedale Molinette Torino), Torino, Italy; 107 Legacy Emanuel Medical Center, Portland, USA; 108 London Health Sciences Centre, London, Canada; 109 Maastricht University Medical Centre, Maastricht, The Netherlands; 110 Manipal Hospital Whitefield, Bengaluru, India; 111 Mar del Plata Medical Foundation Private Community Hospital, Buenos Aires, Argentina; 112 Maroondah Hospital, Ringwood East, Australia; 113 Mater Misericordiae University Hospital, Dublin 7, Ireland; 114 Mayo Clinic College of Medicine, Rochester, USA; 115 McGill University Health Centre, Montreal, Canada; 116 Medical College of Wisconsin (Froedtert Hospital), Milwaukee, USA; 117 Medical University of Vienna, Wien, Austria; 118 Medical Department II, LMU Hospital Munich, Munich, Germany; 119 MedStar Washington Hospital Centre, Washington, USA; 120 Monash University, Clayton, Australia; 121 Mount Sinai Medical Centre, Paranthal, India; 122 Nagoya University Hospital, Nagoya, Japan; 123 National Cardiovascular Center Harapan Kita, Jakarta, Jakarta, Indonesia; 124 National Taiwan University Hospital, Taipei City, Taiwan; 125 Nemours Alfred I duPont Hospital for Children, Wilmington, USA; 126 North Estonia Medical Centre, Wilmington, USA; 127 Northwell Health, New York, USA; 128 Obihiro-Kosei General Hospital, Wilmington, USA; 129 Ochsner Clinic Foundation, Baton Rouge, USA; 130 Ohio State University Medical Centre, Columbus, USA; 131 Oklahoma Heart Institute, Tulsa, USA; 132 Oregon Health and Science University Hospital (OHSU), Portland, USA; 133 Ospedale Di Arco (Trento Hospital), Arco, Italy; 134 Ospedale San Gerardo, Monza, Italy; 135 Ospedale San Paolo, Milano, Italy; 136 Padua University Hospital (Policlinico of Padova), Padova, Italy; 137 Pamela Youde Nethersole Eastern Hospital, Chai Wan, Hong Kong; 138 Papworth Hospitals NHS Foundation Trust, Cambridge, UK; 139 Penn Medicine (Hospital of the University of Pennsylvania), Philadelphia, USA; 140 Persahabatan General Hospital, Jakarta, Indonesia; 141 Perth Childrens Hospital, Nedlands, Australia; 142 Piedmont Atlanta Hospital, Atlanta, USA; 143 Policlinico di S. Orsola, Università di Bologna, Bologna, Italy; 144 Presbyterian Hospital Services, Albuquerque, USA; 145 Princess Margaret Hospital, Toronto, Canada; 146 Prof Dr R. D. Kandou General Hospital - Paediatric, Manado, Indonesia; 147 Prof Dr R. D R. D. Kandou General Hospital - Adult, Manado, Indonesia; 148 Dr Sulianti Saroso Hospital, Jakarta, Indonesia; 149 Providence Saint Johns Health Centre, Santa Monica, USA; 150 Queen Elizabeth Hospital, Pok Fu Lam, Hong Kong; 151 The University of Hong Kong, Pok Fu Lam, Hong Kong; 152 Redcliffe Hospital, Redcliffe, Australia; 153 Rinku General Medical Center (and Senshu Trauma and Critical Care Center), Izumisano, Japan; 154 Rio Hortega University Hospital, Valladolid, Spain; 155 Rochester General Hospital, Rochester, USA; 156 Royal Childrens Hospital, Melbourne, Australia; 157 Royal North Shore Hospital, St Leonards, Australia; 158 Royal Prince Alfred Hospital, Camperdown, Australia; 159 RSUD Soetomo, Jawa Timur, Indonesia; 160 Rush University, Chicago, USA; 161 Saiful Anwar Malang Hospital (Brawijaya University) (Paediatrics), Jawa Timur, Indonesia; 162 Saiful Anwar Malang Hospital (Brawijaya University) (Adult), Jawa Timur, Indonesia; 163 Saiseikai Senri Hospital, Suita, Japan; 164 Saiseikai Utsunomiya Hospital, Utsunomiya, Japan; 165 San Martino Hospital, Genova, Italy; 166 San Pedro de Alcantara Hospital, Cáceres, Spain; 167 Sao Camilo Cura Dars, Fortaleza, Brazil; 168 São João Hospital Centre, Porto, Portugal; 169 Sardjito Hospital (Paediatrics), Yogyakarta, Indonesia; 170 Sentara Norfolk General Hospital, Norfolk, USA; 171 Seoul National University Bundang Hospital, Seongnam-si, South Korea; 172 Severance Hospital, Seongnam-si, South Korea; 173 Shizuoka Childrens Hospital, Shizuoka, Japan; 174 Sinai Health Systems (Mount Sinai Hospital), New York, USA; 175 Siriraj Hospital, Bangkok, Thailand; 176 Sozialmedizinisches Zentrum Süd – Kaiser-Franz-Josef-Spital, Wien, Austria; 177 St Boniface Hospital (University of Mannitoba), Winnipeg, Canada; 178 St Christophers Hospital for Children, Philadelphia, USA; 179 St George Hospital, Mumbai, India; 180 St Marianna Medical University Hospital, Kawasaki, Japan; 181 Stanford University Hospital, Stanford, USA; 182 St Vincents Hospital, Worcester, USA; 183 Tartu University Hospital, Tartu, Estonia; 184 Teine Keijinkai Hospital, Sapporo, Japan; 185 The Alfred Hospital, Melbourne, Australia; 186 The Heart Hospital Baylor Plano, Plano, USA; 187 The Prince Charles Hospital, Chermside, Australia; 188 The University of Kansas Medical Centre, Kansas City, USA; 189 Tohoku Medical and Pharmaceutical University, Sendai, Japan; 190 Tokyo Metropolitan Bokutoh Hospital, Tokyo, Japan; 191 Tokyo Metropolitan Medical Center, Tokyo, Japan; 192 Toronto General Hospital, Turin, Canada; 193 Tokyo Womens Medical University Hospital, Tokyo, Japan; 194 Tufts Medical Centre (and Floating Hospital for Children), Boston, USA; 195 Universitätsklinikum Frankfurt (University Hospital Frankfurt) (Uniklinik), Frankfurt am Main, Germany; 196 Universitätsklinikum Regensburg (Klinik Für Innere Medizin II), Regensburg, Germany; 197 University Airlangga Hospital (Paediatric), Melbourne, Australia; 198 University Hospital Cleveland Medical Centre (UH Cleveland Hospital), Cleveland, USA; 199 University Hospital in Krakow, Kraków, Poland; 200 University Hospitals of Leicester NHS Trust (Glenfield Hospital), Leicester, UK; 201 University of Alabama at Birmingham Hospital (UAB), Birmingham, USA; 202 University of British Columbia, Vancouver, Canada; 203 University of Calgary (Peter Lougheed Centre, Foothills Medical Centre, South Health Campus and Rockyview General Hospital), Calgary, Canada; 204 University of California, San Francisco-Fresno Clinical Research Centre, San Francisco, USA; 205 University of Chicago, Chicago, USA; 206 University of Cincinnati Medical Centre, Cincinnati, USA; 207 University of Florida, Gainesville, USA; 208 University of Iowa, Iowa City, USA; 209 University of Maryland - Baltimore, Baltimore, USA; 210 University of Michigan Medical Center, Ann Arbor, USA; 211 University of Milan, Milano, Italy; 212 University of Nebraska Medical Centre, Omaha, USA; 213 University of Oklahoma Health Sciences Centre (OU), Oklahoma City, USA; 214 University of Utah Hospital, Salt Lake City, USA; 215 Vall dHebron University Hospital, Barcelona, Spain; 216 Washington University in St. Louis/ Barnes Jewish Hospital, Washington, USA; 217 Yokohama City University Medical Center, Yokohama, Japan; 218 COVID-19 Critical Care Consortium, New York, USA; 219 SARIC, Centre for Tropical Medicine and Global Health, University of Oxford, Oxford, UK; 220 Aberdeen Royal Infirmary (Foresterhill Health Campus), Aberdeen, UK; 221 Adam Malik Hospital, Medan, Indonesia; 222 Aichi Childrens Health and Medical Center, Obu, Japan; 223 American Hospital, New York, USA; 224 Anam Korea University Hospital, Seoul, South Korea; 225 Auckland City Hospital, Auckland, New Zealand; 226 Austin Hospital, Heidelberg, Australia; 227 Azienda Ospedaliero Universitaria Parma, Parma, Italy; 228 Banner University Medical Centre, Tucson, USA; 229 Baptist Health Louisville, Louisville, USA; 230 Barts Hospital, London, UK; 231 Billings Clinic, Billings, USA; 232 Brigham and Womens Hospital, Boston, USA; 233 Borgo San Lorenzo Hospital, Rome, Italy; 234 Brighton and Sussex Medical School, Brighton, UK; 235 Brooke Army Medical Centre, Fort Sam Houston, USA; 236 Bundaberg Hospital, Bundaberg Central, Australia; 237 Cairo University Hospital, Cairo, Egypt; 238 Cardio VID, Medellín, Colombia; 239 Careggi Hospital, Florence, Italy; 240 Cedar Park Regional Medical Center, Cedar Park, USA; 241 Cedars-Sinai Medical Centre, Los Angeles, USA; 242 Cengkareng Hospital, Jakarta, Indonesia; 243 Centro Hospitalar de Lisboa, Lisbon, Portugal; 244 Charite-Univerrsitatsmedizi N Berlin, Berlin, Germany; 245 Charles Nicolle University Hospital, Tunis, Tunisia; 246 Childrens Health Ireland (CHI) at Crumlin, Dublin, Ireland; 247 Childrens Hospital Bambino Gesù, Rome, Italy; 248 Childrens Healthcare of Atlanta – Egleston Hospital, Atlanta, USA; 249 Childrens Hospital –Los Angeles, Los Angeles, USA; 250 Christiana Care Health Systems Centre for Heart and Vascular Health, Newark, USA; 251 CHU de Charleroi, Charleroi, Belgium; 252 Chungbuk National University Hospital, Cheongju-si, South Korea; 253 Cincinnati Childrens, Cincinnati, USA; 254 Cipto Mangunkusumo Hospital, Jakarta, Indonesia; 255 Città della Salute e della Scienza Hospital – Turin, Turin, Italy; 256 Clínica Sagrada Família, New York, USA; 257 Clinica Universidad de Navarra, Navarra, Spain; 258 Collaborative Centre Department Cardiac Surgery, UZ Leuven, Leuven, Belgium; 259 Dignity Health Medical Group- Dominican, San Francisco, USA; 260 Dignity Health St. Josephs Hospital and Medical Center (SJHMC), Phoenix, USA; 261 Doernbecher Childrens Hospital, Portland, USA; 262 Donaustauf Hospital, Donaustauf, Germany; 263 Kariadi Hospital Semarang, Jawa Tengah, Indonesia; 264 Suat Seren Chest Diseases and Surgery Practice and Training Centre, İzmir, Turkey; 265 Duke University Hospital (Durham), Durham, USA; 266 Eunpyeung St Marys Hospital, Seoul, South Korea; 267 Fluminense Federal University, Niterói, Brazil; 268 Fundación Clinica Shaio (Shaio Clinic), Bogotá, Colombia; 269 Gdansk Medical University, Gdańsk, Poland; 270 General University Hospital, London, UK; 271 George Washington University Hospital, Washington, USA; 272 Giesinger Medical Centre, Philadelphia, USA; 273 Gunma University Graduate School of Medicine, Maebashi, Japan; 274 Guys and St Thomas NHS Foundation Trust Hospital, London, UK; 275 Hakodate City Hospital, Hakodate, Japan; 276 Hallym University Sacred Heart Hospital, New York, USA; 277 Hamilton General Hospital, Hamilton, Canada; 278 Hasan Sadikin Hospital (Paediatric), Jawa Barat, Indonesia; 279 Heart Institute Ministry of Health of Ukraine, Kyiv, Ukraine; 280 Hervey Bay Hospital, Pialba, Australia; 281 Hospital Clinico de La Pontificia Universidad Catolica, Santiago, Chile; 282 Hospital Clinico de La Universidad de Chile, Santiago, Chile; 283 Hospital de Clínicas de Porto Alegre, Porto Alegre, Brazil; 284 Hospital Felicio Rocho, Porto Alegre, Brazil; 285 Hospital Israelita Albert Einstein, São Paulo, Brazil; 286 Hospital Privado de Comunidad, Buenos Aires, Argentina; 287 Hospital Pro Cardíaco, Rio de Janeiro, Brazil; 288 Hospital Punta de Europa, Algeciras, Spain; 289 Hospital Regional de Valdivia, Algeciras, Spain; 290 Hospital Samaritano Paulista, São Paulo, Brazil; 291 Hospital Santa Catarina, São Paulo, Brazil; 292 Hospital Santa Marta, Lisbon, Portugal; 293 Hospital Sirio Libanes, Lisbon, Portugal; 294 Hospital Universitario de Bellvitge, Barcelona, Spain; 295 Hospital Universitario Esperanza (Universidad Francisco Marroquin), Guatemala City, Guatemala; 296 Hospital Universitario Lucus Augusti, Lugo, Spain; 297 Hyogo Prefectural Kobe Childrens Hospital, Minatojima, Japan; 298 Indiana University Health, Indianapolis, USA; 299 Inselspital University Hospital, Indianapolis, IN USA; 300 Institut Universitaire de Cardiologie et de Pneumologie de Quebec - Universite Laval, Monteral, Canada; 301 Instituto de Cardiologia do Distrito Federal – ICDF, Brasília, Brazil; 302 Instituto de Medicina Integral . Fernando Figueira (IMIP), Recife, Brazil; 303 Instituto Goiano de Diagnostico Cardiovascular (IGDC), Goiânia, Brazil; 304 INTEGRIS Baptist Medical Center, Oklahoma City, USA; 305 Japan Red Cross Maebashi Hospital, Maebashi, Japan; 306 John C Lincoln Medical Centre, Phoenix, USA; 307 John Hunter Hospital, New Lambton Heights City, Australia; 308 Kenyatta National Hospital (KNH), Nairobi, Kenya; 309 King Abdullah Medical City, Manama, Bahrain; 310 King Abdullah Medical City Specialist Hospital, Mecca, Saudi Arabia; 311 King Abdullah Medical Complex, Jeddah, Saudi Arabia; 312 King Salman Hospital NWAF, Riyadh, Saudi Arabia; 313 King Saud Medical City, Riyadh, Saudi Arabia; 314 KK Womens and Childrens Hospital, Singapore, Singapore; 315 KKR Medical Center, New York, USA; 316 Kyung Pook National University Hospital, Daegu, South Korea; 317 Lagos University Teaching Hospital, Lagos, Nigeria; 318 Lung Center of the Philippines, Quezon City, Philippines; 319 Luxembourg Heart Center, Rue Dicks, Luxembourg; 320 M Health Fairview, Saint Paul, USA; 321 Maine Medical Centre (Portland Maine), Portland, USA; 322 Manchester University NHS Foundation Trust - Wythenshawe, Wythenshawe, USA; 323 Massachusetts General Hospital, Boston, USA; 324 Medanta Hospital, Gurgaon, India; 325 Medica Superspecialty Hospital, Kolkata, India; 326 Medical University of South Carolina, Charleston, USA; 327 Mediclinic Parkview Hospital Dubai, Dubai, UAE; 328 Memorial Hermann - Texas Medical Centre, Houston, USA; 329 Memorial Regional Hospital (Hollywood Florida), Hollywood, USA; 330 Mercy Hospital of Buffalo, Buffalo, USA; 331 Mie University Hospital, Tsu, Japan; 332 Mohammed VI University Hospital, Bouskoura, Morocco; 333 National Centre for Infectious Diseases, Singapore, Singapore; 334 National Cerebral and Cardiovascular Center, Osaka, Japan; 335 National Kidney and Transplant Institute, Quezon City, Philippines; 336 National University Hospital, Singapore, Singapore; 337 National University of Comahue, Neuquén, Argentina; 338 Nelson Mandela Childrens Hospital, Johannesburg, South Africa; 339 Nepean Hospital, Kingswood, Australia; 340 Netcare Unitas ECMO Centre, Centurion, South Africa; 341 Nippon Medical School Hospital, Bunkyo, Japan; 342 Northwestern Medicine, Chicago, USA; 343 Norton Childrens Hospital, Louisville, USA; 344 Novant Health (NH) Presbyterian Medical Centre, Charlotte, USA; 345 Ochsner LSA Health Shreveport, Shreveport, USA; 346 Okayama University Hospital, Okayama, Japan; 347 Osaka City General Hospital, Osaka, Japan; 348 Ospedale Gaslini, Genova, Italy; 349 Ospedali Dei Colli, Napoli, Italy; 350 Paracelsus Medical University Nuremberg, Salzburg, USA; 351 Parirenyatwa General Hospital, Harare, Zimbabwe; 352 Pediatric and Neonatal Cardiac Intensive Care at the American University, New York, USA; 353 Pelni Hospital, Jakarta, Indonesia; 354 Penn State Heath S. Hershey Medical Centre, Hershey, USA; 355 Peyton Manning Childrens Hospital, Indianapolis, USA; 356 Policlinico Umberto, Sapienza University of Rome, Rome, Italy; 357 Presbyterian Hospital, New York/Weill Cornell Medical Centre, New York, USA; 358 Prince Mohammed Bin Abdulaziz Hospital, Riyadh, Saudi Arabia; 359 Prince of Wales, London, UK; 360 Providence Sacred Heart Childrens Hospital, Spokane, USA; 361 Queen Elizabeth II University Hospital, Birmingham, UK; 362 Queensland Childrens Hospital, South Brisbane, Australia; 363 Queens University of Belfast, Belfast, UK; 364 Rabat University Hospital, Rabat, Morocco; 365 Radboud University Medical Centre, Nijmegen, The Netherlands; 366 Rambam Hospital, Haifa, Israel; 367 Ramón y Cajal University Hospital, Madrid, Spain; 368 Rapha Medical Centre, New York, USA; 369 Redcliffe Hospital, Melbourne, Australia; 370 Rockhampton Hospital, Rockhampton, Australia; 371 Royal Adelaide Hospital, Adelaide, Australia; 372 Royal Brompton & Harefield NHS Foundation Trust, London, UK; 373 Royal Columbian Hospital, New Westminster, Canada; 374 Royal Infirmary Edinburgh, Edinburgh, UK; 375 Sahlgrenska University Hospital, Göteborg, Sweden; 376 Saiseikai Yokohamashi Tobu Hospital, Yokohama, Japan; 377 Sanglah General Hospital, Bali, Indonesia; 378 Santa Casa de Misericordia de Juiz de Fora, Juiz de Fora, Brazil; 379 Santa Casa de Misericórdia de Vitoria, Vitória, Brazil; 380 Sardjito Hospital, Yogyakarta, Indonesia; 381 Scripps Memorial Hospital La Jolla, San Diego, USA; 382 Seoul National University Childrens Hospital, Seoul, Korea; 383 Seoul National University Hospital, Seoul, Korea; 384 Sheba Medical Center, Ramat Gan, Israel; 385 Shimane University Hospital, Izumo, Japan; 386 Soetomo General Hospital (FK UNAIR), Surabaya, Indonesia; 387 Spectrum Health Western Governors University, Greenville, USA; 388 St. Antonius Hospital, Nieuwegein, The Netherlands; 389 St Jamess University Hospital, Leeds, UK; 390 Swansea Hospital, Swansea, UK; 391 Tan Tock Seng Hospital, Singapore, Singapore; 392 The Catholic University of Seoul St Mary Hospital, Seoul, South Korea; 393 The Christ Hospital, Cincinnati, USA; 394 The Medical Centre Navicent Health, Macon, USA; 395 Thomas Jefferson University Hospital, Philadelphia, USA; 396 Tohoku University, Sendai, Japan; 397 Tuen Mun Hospital, Tuen Mun, Hong Kong; 398 UCLA Medical Centre (Ronald Regan), Los Angeles, USA; 399 Universitätsspital Bern, Universitätsklinik Für Herz- Und Gefässchirurgie, Bern, Switzerland; 400 Universite Libre de Bruxelles, Brussels, Belgium; 401 University Airlangga Hospital (Adult), Surabaya, Indonesia; 402 University de Montreal (Montreal Heart Institute), Montreal, Canada; 403 University Hospital CHLN, Lisbon, Portugal; 404 University Hospital Dubrava, Zagreb, Croatia; 405 University Hospital Leuven, Leuven, Belgium; 406 University Medical Center Groningen, Groningen, The Netherlands; 407 University of Aberta (Mazankowski Heart Institute), Edmonton, Canada; 408 University of Antwerp, Antwerp, Belgium; 409 University of Calgary and Alberta Health Services, Calgary, Canada; 410 University of California at San Diego, San Diego, USA; 411 University of California, Irvine, USA; 412 University of Kentucky Medical Center, New York, USA; 413 University of Missouri, Columbia, USA; 414 University of Pittsburgh Medical Centre, Pittsburgh, USA; 415 University of Rochester Medical Centre (UR Medicine), Rochester, USA; 416 University of South Carolina, Columbia, USA; 417 University of Texas Medical Branch, Galveston, USA; 418 University of the Ryukyus, Nishihara, Japan; 419 University of Washington in Seattle, Seattle, USA; 420 University of Wisconsin & American Family Childrens Hospital, Madison, USA; 421 UT Southwestern, Dallas, USA; 422 UTHealth (University of Texas), Houston, USA; 423 Valley Childrens Hospital (Madera), Madera, USA; 424 Vassar Brothers Medical Center (VBMC), New York, USA; 425 Verona Integrated University Hospital, Verona, Italy; 426 Wahidin Sudirohusodo Hospital, Makassar, Indonesia; 427 WellSpan Health - York Hospital, York, USA; 428 Westmead Hospital, Sydney, Australia; 429 Yale New Haven Hospital, New Haven, USA

**Keywords:** SARS-CoV-2, COVID-19, Neuromuscular blocking agent, Mechanical ventilation, Intensive care unit

## Abstract

**Background:**

The role of neuromuscular blocking agents (NMBAs) in coronavirus disease 2019 (COVID-19) acute respiratory distress syndrome (ARDS) is not fully elucidated. Therefore, we aimed to investigate in COVID-19 patients with moderate-to-severe ARDS the impact of early use of NMBAs on 90-day mortality, through propensity score (PS) matching analysis.

**Methods:**

We analyzed a convenience sample of patients with COVID-19 and moderate-to-severe ARDS, admitted to 244 intensive care units within the COVID-19 Critical Care Consortium, from February 1, 2020, through October 31, 2021. Patients undergoing at least 2 days and up to 3 consecutive days of NMBAs (NMBA treatment), within 48 h from commencement of IMV were compared with subjects who did not receive NMBAs or only upon commencement of IMV (control). The primary objective in the PS-matched cohort was comparison between groups in 90-day in-hospital mortality, assessed through Cox proportional hazard modeling. Secondary objectives were comparisons in the numbers of ventilator-free days (VFD) between day 1 and day 28 and between day 1 and 90 through competing risk regression.

**Results:**

Data from 1953 patients were included. After propensity score matching, 210 cases from each group were well matched. In the PS-matched cohort, mean (± SD) age was 60.3 ± 13.2 years and 296 (70.5%) were male and the most common comorbidities were hypertension (56.9%), obesity (41.1%), and diabetes (30.0%). The unadjusted hazard ratio (HR) for death at 90 days in the NMBA treatment vs control group was 1.12 (95% CI 0.79, 1.59, *p* = 0.534). After adjustment for smoking habit and critical therapeutic covariates, the HR was 1.07 (95% CI 0.72, 1.61, *p* = 0.729). At 28 days, VFD were 16 (IQR 0–25) and 25 (IQR 7–26) in the NMBA treatment and control groups, respectively (sub-hazard ratio 0.82, 95% CI 0.67, 1.00, *p* = 0.055). At 90 days, VFD were 77 (IQR 0–87) and 87 (IQR 0–88) (sub-hazard ratio 0.86 (95% CI 0.69, 1.07; *p* = 0.177).

**Conclusions:**

In patients with COVID-19 and moderate-to-severe ARDS, short course of NMBA treatment, applied early, did not significantly improve 90-day mortality and VFD. In the absence of definitive data from clinical trials, NMBAs should be indicated cautiously in this setting.

**Supplementary Information:**

The online version contains supplementary material available at 10.1186/s13054-022-03983-5.

## Background

Since early 2020, SARS-CoV-2 infections have placed tremendous burden on patients and international healthcare services [1]. A high proportion of diseased patients require hospitalization, and a small subset with severe coronavirus disease 2019 (COVID-19) become critically ill and require invasive mechanical ventilation (IMV) for life-threatening respiratory failure [2–5]. High mortality has been reported in this subpopulation [4, 6–9], irrespective of survival benefits from established treatments, such as corticosteroids and IL-6 receptor antagonists [10, 11].

Neuromuscular blocking agents (NMBAs) have been commonly used for acute respiratory distress syndrome (ARDS) [12, 13] to reduce patient–ventilator asynchrony and—in the context of severely damaged and poorly compliant lungs—to improve oxygenation, while minimizing the work of breathing and risks of barotrauma. International studies have confirmed the use of NMBAs in up to 26% of ARDS patients [14]. Yet, conflicting results were provided by large randomized clinical trials, such as the ACURASYS [15] and ROSE trials [16], and indication, efficacy, and safety on the use of NMBAs in such patients remain uncertain.

The severe respiratory failure associated with COVID-19 has been described as a form of ARDS, and the potential benefits of supportive treatments have largely been extrapolated from evidence in non-COVID-19-related ARDS. Thus, many COVID-19 patients have been treated with NMBAs [17, 18], and in some areas even shortage of these medications has been reported [19], especially during the early phase of the pandemic. To the best of our knowledge, no international studies have clearly elucidated the effects of NMBAs on mortality in COVID-19 patients. Interestingly, in a multicenter observational study, Courcelle et al. [20] reported that the duration of NMBA treatment in this population was often longer than 48 h and not associated with shorter duration of IMV.

To further delineate the role of NMBA in ventilated COVID-19 patients, data extracted from the multicenter registry of the international COVID-19 Critical Care Consortium incorporating the ExtraCorporeal Membrane Oxygenation for 2019 novel Coronavirus Acute Respiratory Disease (COVID-19–CCC/ECMOCARD) were examined. Our hypothesis was that in patients with microbiologically confirmed COVID-19 and moderate-to-severe ARDS, no significant difference in 90-day hospital mortality was to be found between populations who received or not early NMBAs. Thus, the primary goal of this study was to identify difference in 90-day mortality, through propensity score (PS)-adjusted analysis, between patients who received or not a short course of NMBAs, within 48 h from commencement of IMV.

## Methods

### Study design

This was a comparative study in which mechanically ventilated COVID-19 patients with moderate-to-severe ARDS, who received an early short course of NMBAs, were compared with patients who did not receive NMBA or underwent NMBA treatment only on the day of commencement of IMV to explore the impact on in-hospital mortality during a follow-up period up to 90 days. Of note, the STROBE (Strengthening the Reporting of Observational Studies in Epidemiology) guidelines were used to ensure the reporting of this observational study [21].

### Study population and settings

#### Inclusion criteria

We studied a convenience sampling of patients (≥ 16 years old) with microbiologically confirmed *SARS-CoV-*2 infection, via rapid nucleic acid amplification test (NAAT) or antigen tests, who were admitted to an intensive care unit (ICU) from February 1, 2020, through October, 31, 2021, at any of the COVID-19 Critical Care Consortiums participating hospitals. In addition, patients were selected if they presented with moderate-to-severe ARDS, defined by a ratio of the partial pressure of arterial oxygen to the fraction of inspired oxygen of < 150 mm Hg [16], within 48 h from commencement of IMV.

#### Exclusion criteria

We excluded patients with unrecorded date of commencement of IMV or who were transferred from other institutions, after commencement of IMV. In addition, we excluded from the primary analysis all patients with continuous use of NMBA for periods longer than 3 days, and intermittent use of NMBA within a 3-day period of treatment.

### Variables, data sources, and measurements

Data on demographics, comorbidities, clinical symptoms, and laboratory results were collected by clinical and research staff of the participating ICUs in an electronic case report form [22]. Details of respiratory and hemodynamic support, physiological variables, and laboratory results were collected daily, with worst daily values recorded preferentially. Duration of IMV, length of ICU and hospital stays, and hospital mortality were also recorded.

### Study groups

In line with previous therapeutic protocols applied in patients with moderate-to-severe ARDS [15, 16], we aimed to appraise a short course of NMBAs, applied early during the course of IMV. Thus, patients were assigned to the following study groups.

#### NMBA treatment

Use of NMBA treatment for at least 48 h and up to 3 consecutive days, initiated early, within 48 h from commencement of IMV.

#### Control

No use of NMBAs or administration only on the day IMV was commenced.

Of note, the time of first NMBAs administration was not recorded on the case report form; thus, the treatment group was designed to include up to 3 consecutive days of NMBAs exposure to ensure that at least 2 days of full treatment were achieved in most of the patients, even if treatment commenced during the night or a single NMBA dose was given upon endotracheal intubation. In addition, patients who were tracheally intubated and transferred from institutions not collaborating with the COVID-19-CCC/ECMOCARD were excluded from the analysis to avoid enrolment of subjects who might have received NMBA prior to study monitoring. Recording of NMBA use was censored at 28 days from ICU admission, or upon discharge from the ICU or death, whichever occurred first.

### Outcomes

The primary objective was comparison between groups in 90-day in-hospital mortality, through PS matching analysis. Secondary objectives were the numbers of ventilator-free days (days since successful weaning from mechanical ventilation) between day 1 and day 28 and between day 1 and day 90 in the PS-matched population. VFD was calculated as follows: VFDs = 0 if subject died in hospital within 28 or 90 days of mechanical ventilation; VFDs = 28 or 90 − *x* if successfully liberated from ventilation *x* days after initiation; VFDs = 0 if the subject was mechanically ventilated for more than 28 or 90 days.

### Data source

We analyzed the COVID-19-CCC/ECMOCARD study dataset. COVID-19-CCC/ECMOCARD is an international, multicenter, observational cohort study ongoing in 354 hospitals across 54 countries (Additional file 1: Appendix). The full study protocol has been published elsewhere [22]. The COVID-19-CCC/ECMOCARD observational study was reviewed under the National Mutual Acceptance scheme by the Alfred Health Human Research Ethics Committee on February 27, 2020. The ethics committee certified that the study protocol met the requirements of the National Statement on Ethical Conduct in Human Research (2007), granting approval on March 2, 2020. In addition, in accordance with the Office of the Health Services Commissioners Statutory Guidelines on Research issued for the purposes of Health Privacy Principles, the Alfred Health Human Research Ethics Committee granted a waiver of consent for the collection, use, and disclosure of participants health and personal information. Subsequently, the protocol was approved by all international participating hospitals prior to data collection and waiver of consent was granted in all centers.

### Data management and quality

The COVID-19 Consortium collaborates with the International Severe Acute Respiratory and Emerging Infection Consortium (ISARIC) group [23] and their Short PeRiod IncideNce sTudy of Severe Acute Respiratory Infection (SPRINT-SARI) [24] project. De-identified patient data are recorded by data collectors at each collaborating site via the REDCap (Vanderbilt/NIH/NCATS UL1 TR000445 v.10.0.23) electronic data capture tool, using instances hosted at the University of Oxford (UK), University College Dublin (Ireland), Monash University (Australia), and The University of Queensland (Australia). A detailed data dictionary was provided to all sites to assist in data collection. In addition, biweekly drop-in data sessions were scheduled to assist with all queries that data collectors might have. Importantly, the database quality audits of the COVID-19 Consortium dataset are a continuing and intensive process encompassing (1) data cleaning rules; (2) checks for outliers; (3) filtering rules setup during the initial development of the case report form, which was periodically monitored/adjusted; and (4) data completeness checks. Finally, in the case any issue was detected during monitoring of data quality, or statistical analysis, these matters were pursued to address any data collection/process limitation in a timely manner, often including follow-up with the site that entered the data for value verification and correction where appropriate.

### Statistical analyses

Categorical data are presented as frequency and percentage, while continuous data as mean and standard deviation (SD; normally distributed) and median and interquartile range (IQR; non-normally distributed). Normality of continuous data was reviewed via visual inspection of histograms. Bivariate comparisons between patients receiving NMBA treatment and controls were compared using Fishers exact test, Students unpaired t test, or the Mann–Whitney *U* test for categorical, normally, and non-normally distributed continuous variables, respectively.

Analyses were performed to examine the relationship between the primary outcome—mortality censored at 90 days post-commencement of IMV—and NMBA treatment. Patients entered the study analysis once they commenced IMV. As an imbalance in several characteristics at ICU admission between the study groups was noted, propensity score (PS) matching analysis was undertaken to balance these patient characteristics. Matching was undertaken, in preference to other techniques involving propensity scores, to best mimic the results of a randomized controlled trial. Baseline features considered potential variables for inclusion in the PS calculation were demographic and clinical characteristics with either a documented relationship with mortality or a baseline imbalance. Variables with more than 5% missing data were not considered. Based on the available literature on risk factors associated with mortality in severe COVID-19, the following covariates were included during PS modeling: age, sex, region (reference group: North America), time from symptom onset to hospital admission and baseline comorbidities (PaO_2_/FiO_2_ within 48 h from commencement of IMV, hypertension, chronic cardiac disease, chronic kidney disease, obesity). Matched cohorts were constructed based on the logit of the PS using nearest-neighbor matching with a caliper width of 0.2 and replacement to promote better balance, and accounting for the estimation of the standard errors. Rubins *B* [25] (a summation measure of bias with value < 25% indicating adequate balance) and Rubins *R* (0.5–2.0 indicating appropriate balancing) statistics were initially calculated to assess the success of the matching (i.e., if the resulting matched cohort had balanced characteristics). Standardized mean differences for all covariates, before and after matching, were then estimated, with an absolute difference of 10% or greater considered indicative of imbalance [26, 27].

Following PS matching, survival curves were generated for the matched sample to compare patients undergoing NMBA treatment against control. Finally, multivariable Cox regression models were constructed to assess the effect of NMBA treatment on outcome—incorporating robust estimators of variance to account for matched observations—as well as investigating effect modification on the association between mortality and NMBA by concomitant use of corticosteroids and respiratory failure severity. Covariates considered for inclusion in the multivariable Cox regression were laboratory results upon admission, treatments received and ventilator settings; covariates with known relationship with mortality in COVID-19 patients available in this dataset with less than 5% missing data were included in the final model. Where potential covariates were correlated, the most relevant clinical variable was chosen for inclusion in the model to avoid multicollinearity and the violation of model assumptions. Continuous covariates were standardized by subtracting the mean and dividing by the standard deviation to facilitate meaningful interpretation of the hazard ratios. Multiple imputation was not undertaken, and complete case analysis was performed [28]. Assumptions of the Cox regression model, particularly the proportional hazards assumption, were evaluated using test of the Schoenfeld residuals, as well as graphical exploration of the log–log plot; the proportional hazards assumption was met. Competing risk regression was performed to assess the secondary outcomes of VFDs at 28 and 90 days post-starting of IMV [29].

#### Sensitivity analyses

Considering the observational nature of our report and potential limitations related to PS matching, we also balanced baseline patient characteristics by weighting each individual in the analysis, by the inverse probability of receiving exposure to NMBAs (inverse probability of treatment weighting [IPTW]) [30]. In addition, as the dataset did not report exact NMBA administration practice, the following sensitivity analyses were conducted to confirm the results of the primary analysis: (1) in patients with clinically suspected and microbiologically confirmed COVID-19, with the treatment group defined as per the primary analysis; and in the treatment group only including patients who received NMBAs (2) continuously for 2 days; (3) continuously for 3 days; and (4) continuously for more than 3 days.

Of note, a post hoc power calculation was undertaken, as a convenience sample was being used. In the unmatched cohort, assuming type I error of 0.05, as well as the rate of primary outcome and study group size as reported in the results, the analysis had 98% power, adequate to undertake and report on the analyses of interest. Data were analyzed using StataSE version 17.0 (StataCorp Pty Ltd., College Station, Texas). Any two-tailed *p* value less than 0.05 was considered significant. No correction was made for multiple comparisons.

## Results

### Study population

**Fig. 1:**
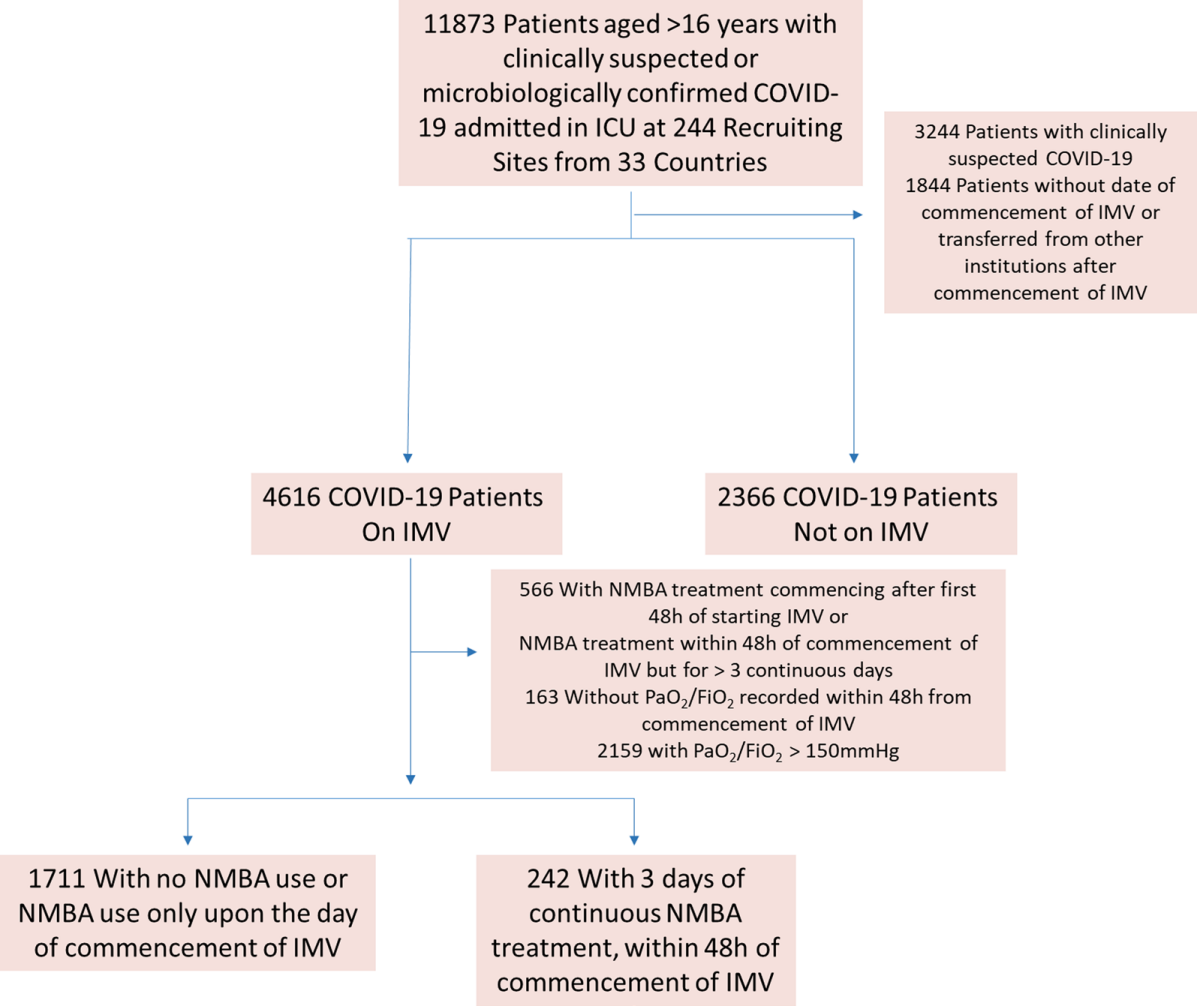
Flow of patient enrolment by the censor date of October 31, 2021. MV, mechanical ventilation; NMBA, neuromuscular blocking agents

**Fig. 2:**
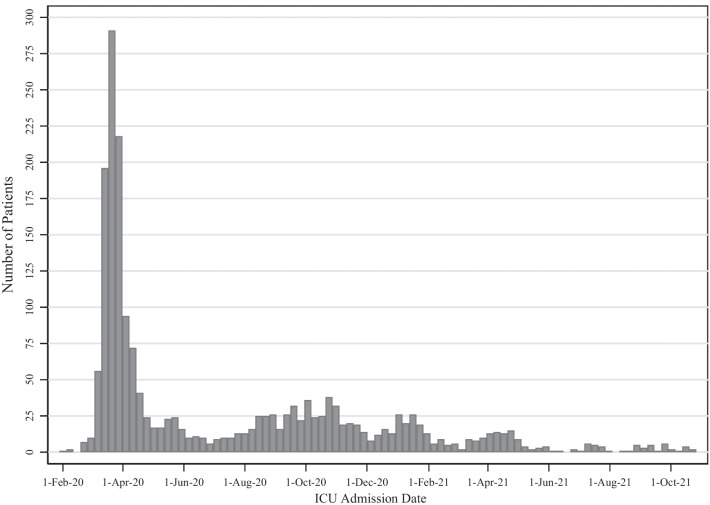
Number of patients per week of intensive care unit (ICU) admission is depicted

There were 11,873 patients with microbiologically confirmed, or suspected, COVID-19 admitted to 244 hospitals between February 1, 2020, and October 31, 2021. After excluding 3244 patients because of lack of microbiological confirmation of SARS-CoV-2 infection and 1844 patients who were transferred from non-collaborating centers, or transferred out while still on mechanical ventilation, 4616 patients who were on IMV were included in the primary analysis (Fig. 1). Figure 2 shows enrolment rate of those mechanically ventilated patients by date of ICU admission. A matched cohort based on propensity scores was generated, with 210 patients who underwent NMBA treatment and 210 patients as control group. Overall, baseline characteristics were balanced in the propensity score-matched cohort. The mean age was 60.3 years (standard deviation [SD] 13.2 years) (Table 1) and 296 (70.5%) were male. The mean acute physiology and chronic health evaluation (APACHE) II score was 19.5 (SD 11.6; *N* = 117). In the NMBA treatment and control group, respiratory system compliance was 32.5 mL/cmH_2_O (SD 12.6) and 33.9 (SD 12.2), respectively, and driving pressure was 22.7 cmH_2_O (SD 7.5) and 22.4 (SD 8.6). The most common comorbidities among patients in the PS-matched cohort were hypertension (239, 56.9%), obesity (172, 41.1%), and diabetes (125, 30.0%).

**Table 1:** Demographic and clinical characteristics upon intensive care unit admission of patients who received or did not receive NMBA treatment

	Unmatched cohort (*N* = 1953)			Propensity score-matched cohort (*N* = 420)	
Parameter	Control (*N* = 1711)		NMBA treatment (*N* = 242)	Control (*N* = 210)	NMBA treatment (*N* = 210)
Age (years) mean (SD)	61.8 (12.3)		58.9 (12.2)	61.2 (14.2)	59.4 (12.1)
*Age (years) n (%)*					
< 50	268 (15.7%)		50 (20.7%)	42 (20.0%)	40 (19.0%)
50–59	366 (21.4%)		66 (27.3%)	45 (21.4%)	56 (26.7%)
60–69	578 (33.8%)		80 (33.1%)	57 (27.1%)	73 (34.8%)
70–79	448 (26.2%)		43 (17.8%)	59 (28.1%)	39 (18.6%)
≥ 80	51 (3.0%)		3 (1.2%)	7 (3.3%)	2 (1.0%)
Male *n* (%)	1,196 (70.0%)		175 (72.3%)	144 (68.6%)	152 (72.4%)
Duration of symptom onset to hospital admission (days) median (IQR)	6.0 (4.0–9.0)		7.0 (4.0–9.0)	6.0 (4.0–9.0)	7.0 (3.0–9.0)
Duration of symptom onset to ICU admission (days) median (IQR)	11.0 (8.0–15.0)		10.0 (7.0–13.0)	10.0 (8.0–15.0)	10.0 (7.0–13.0)
Duration of symptom onset to first use of mechanical ventilation (days) median (IQR)	11.0 (8.0–15.0)		10.0 (7.0–13.0)	10.0 (8.0–15.0)	10.0 (7.0–13.0)
*Ethnicity*					
Aboriginal	5 (0.3%)		0 (0.0%)	3 (1.4%)	0 (0%)
Arab	59 (3.4%)		6 (2.5%)	8 (3.8%)	4 (1.9%)
Black	53 (3.1%)		21 (8.7%)	11 (5.2%)	20 (9.5%)
East Asian	18 (1.1%)		5 (2.1%)	7 (3.3%)	5 (2.4%)
Latin American	108 (6.3%)		31 (12.8%)	25 (11.9%)	27 (12.9%)
South Asian	75 (4.4%)		9 (3.7%)	8 (3.8%)	9 (4.3%)
West Asian	5 (0.3%)		1 (0.4%)	1 (0.5%)	1 (0.5%)
White	163 (9.5%)		54 (22.3%)	18 (8.6%)	48 (22.9%)
Mixed	11 (0.6%)		8 (3.3%)	4 (1.9%)	5 (2.4%)
Other	22 (1.3%)		10 (4.1%)	1 (0.5%)	10 (4.8%)
Missing	1192 (69.7%)		97 (40.1%)	124 (59.0%)	81 (38.6%)
*Continent*					
Africa *n* (%)	19 (1.1%)		18 (7.4%)	14 (6.7%)	14 (6.7%)
Asia *n* (%)	148 (8.6%)		19 (7.9%)	12 (5.7%)	18 (8.6%)
Europe *n* (%)	1237 (72.3%)		135 (55.8%)	121 (57.6%)	114 (54.3%)
North America *n* (%)	175 (10.2%)		43 (17.8%)	42 (20.0%)	43 (20.5%)
Oceania *n* (%)	30 (1.8%)		7 (2.9%)	3 (1.4%)	5 (2.4%)
South/Central America *n* (%)	102 (6.0%)		20 (8.3%)	18 (8.6%)	16 (7.6%)
Healthcare or laboratory worker *n* (%)	42 (2.6%)		10 (4.5%)	8 (4.0%)	9 (4.7%)
*Comorbidities*					
Smoking *n* (%)	525 (31.0%)		76 (32.1%)	62 (29.5%)	65 (31.4%)
Obesity* *n* (%)	622 (36.7%)		97 (40.8%)	88 (41.9%)	84 (40.2%)
Hypertension *n* (%)	916 (53.9%)		125 (52.7%)	123 (58.6%)	116 (55.2%)
Chronic cardiac disease *n* (%)	227 (13.4%)		21 (8.9%)	17 (8.1%)	19 (9.0%)
Diabetes *n* (%)	502 (29.8%)		65 (27.9%)	65 (31.0%)	60 (29.1%)
Malignant neoplasm *n* (%)	66 (3.9%)		8 (3.4%)	9 (4.3%)	8 (3.8%)
Chronic pulmonary disease *n* (%)	171 (10.1%)		30 (12.6%)	32 (15.2%)	27 (12.9%)
Severe liver disease *n* (%)	30 (1.8%)		6 (2.5%)	4 (1.9%)	6 (2.9%)
Chronic kidney disease *n* (%)	152 (9.0%)		12 (5.0%)	12 (5.7%)	12 (5.7%)
BMI mean (SD)	30.2 (6.4)		30.9 (6.8)	30.3 (6.2)	30.6 (6.6)
*Severity of illness*					
APACHE II mean (SD)	18.5 (10.5)		17.8 (11.0)	21.2 (12.3)	18.3 (11.0)
SOFA mean (SD)	6.0 (3.9)		5.3 (3.5)	6.1 (4.7)	5.4 (3.6)
*Laboratory results upon ICU admission*					
WBC count (10*3/µL) median (IQR)		9.6 (6.1–13.0)	8.8 (7.1–11.7)	10.0 (6.4–14.1)	8.7 (7.1–11.4)
Lymphocyte count (10*3/µL) median (IQR)		0.7 (0.5–1.1)	0.8 (0.5–1.0)	0.8 (0.5–1.2)	0.8 (0.5–1.0)
Neutrophils/lymphocyte ratio median (IQR)		9.9 (5.8–17.9)	10.6 (6.2–15.8)	9.8 (5.5–17.8)	10.4 (6.0–15.2)
Temperature (°C) mean (SD)		37.3 (1.1)	37.4 (1.1)	37.2 (1.0)	37.4 (1.0)
Creatinine (mg/dL) median (IQR)		0.9 (0.7–1.2)	0.8 (0.7–1.1)	1.0 (0.7–1.4)	0.8 (0.7–1.1)
C-reactive protein level (mg/dL) median (IQR)		104.8 (30.0–192.0)	86.3 (22.6–185.4)	126.3 (40.9–228.6)	82.8 (20.8–172.1)
D-dimer (mcg/mL) median (IQR)		0.9 (0.5–2.2)	1.0 (0.5–2.3)	1.0 (0.6–2.9)	1.1 (0.5–2.3)
Lactate (mmol/L) median (IQR)		1.5 (1.1–2.1)	1.6 (1.1–2.1)	1.6 (1.1–2.0)	1.5 (1.1–2.1)
Ferritin (ng/mL) median (IQR)		2.9 (1.4–4.8)	2.8 (1.7–5.6)	3.4 (1.7–5.9)	3.2 (1.7–5.6)
IL-6 (ng/L) median (IQR)		124.7 (51.2–268.0)	79.4 (28.9–108.2)	83.0 (47.8–173.3)	75.9 (26.3–100.1)

Demographic and clinical characteristics upon intensive care unit admission of patients who received or did not receive neuromuscular blocking agents (NMBA). NMBA treatment was defined as at least 2 days of continuous use of NMBAs or up to 3 days, within 48 h from commencement of IMV

IMV, invasive mechanical ventilation; BMI, body mass index; APACHE II, acute physiology and chronic health evaluation; SOFA, sequential organ failure assessment; WBC, white blood cells; IL, interleukin

### NMBA therapy

In the full unmatched cohort, 180 (74.4%) patients received NMBAs for 48 h and 62 (25.6%) for 3 days, while in the control group, 1151 (67.3%) patients received NMBAs only on the day IMV started, and 560 (32.7%) never received NMBAs. In the PS-matched cohort, NMBA treatment was noted in 210 patients. Early use of NMBAs for 48 h was reported in 160 (76.2%) of the patients, while 50 (23.8%) patients received NMBAs for 3 days. In the matched control group, patients never received NMBAs, not even upon the day IMV started. NMBA was provided on average 1 day after ICU admission (*N* = 207; IQR 0–2 days). The median length from commencement of IMV to initiation of NMBA therapy was 0 days (IQR 0–0 days).

### ICU management

In the PS-matched cohort, as summarized in Table 2, PaO_2_/FiO_2_ was 88.6 (SD 29.7) vs 86.0 (SD 30.7), in NMBA treatment and control group, respectively. Upon IMV commencement, patients who received NMBA treatment presented PaCO_2_ of 51.0 mmHg (SD 13.7) vs 48.0 mmHg (SD 15.5) in those who did not. Positive end-expiratory pressure in those undergoing NMBA treatment or not was 12.8 cmH_2_O (SD 3.3) vs 11.9 cmH_2_O (SD 3.1), respectively. As for adjunctive therapies, extracorporeal membrane oxygenation, renal replacement therapy, and recruitment maneuvers were provided more frequently to patients who received NMBA treatment (Table 3). Pneumothorax occurred in 21 (10.4%) and 19 (9.6%) of the patients undergoing NMBA treatment or not, respectively.

**Table 2:** Gas exchange and level of ventilatory support within 24 h of commencement of IMV in patients who received or did not receive neuromuscular blocking agents (NMBAs)

	Unmatched cohort (*N* = 1953)		Propensity score-matched cohort (*N* = 420)	
Parameter	Control (*N* = 1711)	NMBA treatment (*N* = 242)	Control (*N* = 210)	NMBA treatment (*N* = 210)
pH mean (SD)	7.4 (0.1)	7.3 (0.1)	7.3 (0.1)	7.3 (0.1)
FiO_2_ (mmHg) mean (SD)	76.1 (21.9)	77.3 (21.9)	81.0 (20.8)	77.5 (22.0)
PaO_2_/FiO_2_ (mmHg) mean (SD)	98.1 (31.1)	88.5 (29.3)	86.0 (30.7)	88.6 (29.7)
PaCO_2_ (mmHg) mean (SD)	48.7 (13.5)	50.9 (13.9)	48.0 (15.5)	51.0 (13.7)
Tidal volume (ml/PBW) mean (SD)	7.1 (1.4)	6.9 (1.4)	7.4 (1.6)	6.8 (1.4)
Respiratory system compliance (mL/cmH_2_O) mean (SD)	33.8 (11.9)	32.9 (12.7)	33.9 (12.2)	32.5 (12.6)
Plateau pressure (cmH_2_O) mean (SD)	25.4 (5.7)	26.1 (5.1)	25.0 (5.9)	26.2 (5.0)
Driving pressure (cmH_2_O) mean (SD)	22.8 (7.4)	22.1 (7.6)	22.4 (8.6)	22.7 (7.5)
Respiratory rate (breaths/min) mean (SD)	23.8 (7.0)	24.5 (7.1)	24.7 (7.4)	24.8 (7.3)
PEEP level (cmH_2_O) mean (SD)	12.0 (3.0)	12.8 (3.3)	11.9 (3.1)	12.8 (3.3)
Heart rate (beats/min) mean (SD)	89.1 (27.4)	93.9 (29.0)	90.9 (30.5)	95.5 (28.4)
Mean arterial pressure (mmHg) mean (SD)	80.9 (19.8)	77.3 (21.6)	80.2 (20.2)	76.7 (21.7)

NMBA treatment was defined as at least 2 days of continuous use of NMBAs or up to 3 days, within 48 h from commencement of IMV

IMV, invasive mechanical ventilation; FiO_2_, inspiratory fraction of oxygen; PaO_2_/FiO_2_, ratio between arterial partial pressure of oxygen and inspiratory fraction of oxygen; PaCO_2_, arterial partial pressure of carbon dioxide; PBW, predicted body weight; PEEP, positive end-expiratory pressure

**Table 3:** Intensive care unit clinical management in patients who received or did not receive neuromuscular blocking agents (NMBAs)

	Unmatched cohort (*N* = 1953)		Propensity score-matched cohort (*N* = 420)	
Parameter	Control (*N* = 1711)	NMBA treatment (*N* = 242)	Control (*N* = 210)	NMBA treatment (*N* = 210)
Vasopressor/Inotropic support *n* (%)	1556 (90.9%)	197 (81.4%)	180 (85.7%)	175 (83.3%)
Antibiotics *n* (%)	1629 (97.4%)	219 (96.5%)	194 (94.2%)	192 (96.5%)
Any antiviral *n* (%)	1399 (82.8%)	160 (70.5%)	154 (73.7%)	142 (71.7%)
Remdesivir *n* (%)	270 (16.1%)	38 (16.2%)	39 (18.8%)	34 (16.6%)
Use of corticosteroids (%)	364 (21.3%)	48 (19.8%)	48 (22.9%)	40 (19.0%)
Continuous renal replacement therapy *n* (%)	16 (0.9%)	11 (4.5%)	1 (0.5%)	10 (4.8%)
Vasoactive drugs *n* (%)	1483 (89.6%)	185 (81.1%)	178 (86.8%)	163 (81.5%)
Cardiac-assist devices *n* (%)	1 (0.1%)	4 (1.7%)	0 (0.0%)	3 (1.4%)
ECMO *n* (%)	132 (7.7%)	36 (14.9%)	14 (6.7%)	35 (16.7%)
Prone positioning *n* (%)	148 (8.6%)	52 (21.5%)	22 (10.5%)	46 (21.9%)
Use of iNO *n* (%)	2 (0.1%)	4 (1.7%)	1 (0.5%)	4 (1.9%)
Use of recruitment maneuvers *n* (%)	11 (0.6%)	20 (8.3%)	3 (1.4%)	19 (9.0%)
Pneumothorax *n* (%)	208 (12.4%)	22 (9.6%)	19 (9.6%)	21 (10.4%)
Duration of mechanical ventilation (days) median (IQR)	2 (2–4)	4 (3–13)	2 (2–10)	4 (3–13)
Duration of ICU stay (days) median (IQR)*	19 (10–34)	16 (8–27)	16 (8–29)	16 (8–27)
Time from ICU admission to death (days) median (IQR)	12 (6–23)	11 (5–18)	8 (2–22)	11 (4–19)
Time from commencement of MV to death (days) median (IQR)	9 (4–20)	10 (2–16)	5 (1–17)	9 (2–18)

NMBA treatment was defined as at least 2 days of continuous use of NMBAs or up to 3 days, within 48 h from commencement of IMV

IMV, invasive mechanical ventilation; ECMO, extracorporeal membrane oxygenation; iNO, inhaled nitric oxide

### Primary outcome: 90-day in-hospital mortality

**Fig. 3:**
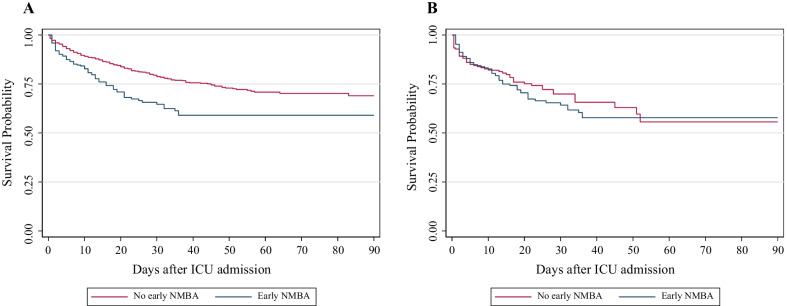
Unadjusted Kaplan–Meier event curves for in-hospital mortality from commencment of invasive mechanical ventilation to 90 days.** A** Before propensity score matching, 90-day ICU Kaplan-Meier curves differed between patients undergoing up to three-day NMBA therapy, within 48 hours from commencement of IMV, in comparison with those who did not (*N* = 1953,* p* < 0.001).** B** After propensity score matching, no difference in survival between patients undergoing NMBA therapy in comparison with those who did not was found (*N* = 420, due to equally sized cohorts post propensity score matching,* P* = 0.537).* NMBA* neuromuscular blocking agent,* ICU* intensive care unit

**Fig. 4:**
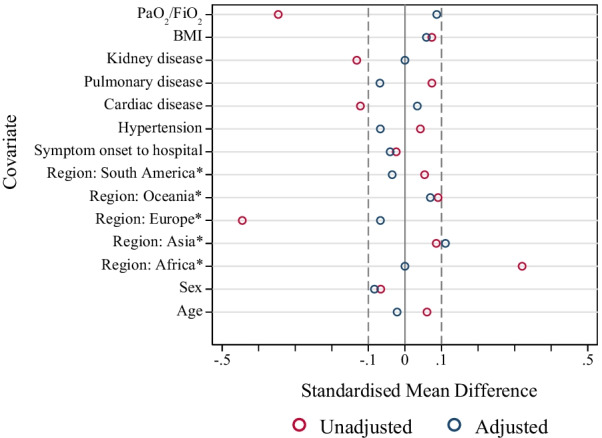
Standard mean difference of key parameters before and after propensity score matching. *Compared with North America

The Kaplan–Meier (Fig. 3A) (log rank test *p* < 0.001) and the Cox regression model (unadjusted HR 1.78; 95% CI 1.38, 2.30; *p* < 0.001) confirmed that NMBA treatment was associated with increased mortality risk. The PS-matched cohort analysis comprised 210 patients in the NMBA treatment group vs 210 controls (using replacements). The summaries of balance for unmatched and matched critical parameters are depicted in Fig. 4. Kaplan–Meier curves (Fig. 3B) (log rank *p* = 0.537) showed no effect of NMBA treatment on mortality, which was also corroborated by the Cox regression model (unadjusted HR = 1.12, 95% CI 0.79, 1.59, *p* = 0.534). The lack of association with 90-day mortality was consistent after adjusting for smoking habit and critical therapeutic covariates (adjusted HR 1.07; 95% CI 0.72, 1.61, *p* = 0.729) (Table 4). In subgroup analyses, PaO_2_/FiO_2_ and the concomitant use of corticosteroids did not alter negative NMBA association with mortality.

**Table 4:** 90-day mortality in patients with coronavirus disease 2019 identified by Cox proportional hazards regression model, considering neuromuscular blocking agents (NMBAs) treatment as at least 2 days of continuous use of NMBAs and up to 3 days, within 48 h from commencement of IMV

	Propensity score-matched cohort^a^ (*N* = 420)		
Variable	Adjusted hazard ratio for 90-day mortality^	95% CI	p value
NMBA treatment	1.07	0.72, 1.61	0.729
NMBA treatment and corticosteroids	1.09	0.39, 3.05	0.872
*NMBA treatment per PaO*_*2*_*/FiO*_*2*_* Strata within 24 h from Commencement of IMV*			
< 100	0.70	0.28, 1.74	0.445
100–149	(Reference)	–	–

^a^Post-propensity score matching, also adjusting for smoking, use of antibiotics, antivirals, corticosteroids, renal replacement therapy, ECMO, and prone positioning. *N* = 420, due to equally sized cohorts post-propensity score matching

IMV, invasive mechanical ventilation; NMBA, neuromuscular blocking agents; PaO_2_/FiO_2_, ratio between arterial partial pressure of oxygen and inspiratory fraction of oxygen

Sensitivity analyses confirmed the lack of association between NMBA treatment and 90-day mortality, when inverse probability weighting analysis was applied (adjusted HR 1.28; 95% CI 0.89, 1.84; *p* = 0.187) and in the analysis of the cohort of patients with clinically suspected and microbiologically confirmed COVID-19 (adjusted HR 1.44; 95% CI 0.99, 2.09, *p* = 0.055) (Table 5). In addition, when analyses were restricted to only 2 days (Table 6) or 3 days of NMBA treatment (Table 7), similar insignificant effect on 90-day mortality was found. Conversely, sensitivity analysis exploring continuous NMBA treatment beyond 3 days (Table 8) showed increased risk of 90-day mortality (adjusted HR 1.73, 95% CI 1.22, 2.37, *p* = 0.001). In this context, the reported median duration of NMBA treatment was 6 days (IQR 5–10).

**Table 5:** NMBA treatment versus controls in patients with clinically suspected and microbiologically confirmed COVID-19 on 90-day in-hospital mortality

	Propensity score-matched cohort (*N* = 792)		
90-day mortality from ICU admission	Control (*N* = 396)	NMBA treatment (*N* = 396)	Total (*N* = 792)
Survived	334 (84.3%)	314 (79.3%)	648 (81.8%)
Died	62 (15.7%)	82 (20.7%)	144 (18.2%)
Total	396	396	792

Prior to propensity score matching, neuromuscular blocking agent (NMBA) treatment, defined as at least 2 days of continuous use of NMBAs or up to 3 days, within 48 h from commencement of invasive mechanical ventilation, was associated with mortality (unadjusted Cox regression; HR 1.33, 95% CI 1.06, 1.66, *p* = 0.015). After propensity score matching, 396 patients who received NMBA treatment were matched with 396 controls (using replacement). Using the propensity score-matched cohort and adjusting for covariates listed in Table 4, NMBA therapy was not associated with 90-day mortality (adjusted HR 1.44, 95% CI 0.99, 2.09, *p* = 0.055)

**Table 6:** Impact of NMBA treatment (defined by 2 days of continuous use of NMBAs within 48 h from commencement of mechanical ventilation) vs controls on 90-day in-hospital mortality

	Propensity score-matched cohort (*N* = 320)		
90-day mortality from ICU admission	Control (*N* = 160)	NMBA treatment (*N* = 160)	Total (*N* = 320)
Survived	117 (73.1%)	112 (70.0%)	229 (72.6%)
Died	43 (26.9%)	48 (30.0%)	91 (28.4%)
Total	160	160	320

Prior to propensity score matching, neuromuscular blocking agent (NMBA) treatment, defined as 2 days of continuous use of NMBAs, within 48 h from commencement of invasive mechanical ventilation, was associated with 90-day mortality (unadjusted Cox regression; HR 1.65, 95% CI 1.22, 2.23, *p* = 0.001). After propensity score matching, 160 patients who received NMBA treatment were matched with 160 controls (using replacement). Using the propensity score-matched cohort and adjusting for covariates as per Table 4, NMBA therapy was not associated with 90-day mortality (adjusted HR 1.15, 95% CI 0.74, 1.78, *p* = 0.524)

**Table 7:** Prior to propensity score matching, neuromuscular blocking agent (NMBA) treatment, defined as 3 days of continuous use of NMBAs, within 48 h from commencement of invasive mechanical ventilation, was associated with 90-day mortality (unadjusted Cox regression; HR 2.13, 95% CI 1.40, 3.24,* P* < 0.001). After propensity-score matching, 50 patients who received NMBA treatment were matched with 50 controls (using replacement). Using the propensity score-matched cohort (and adjusting for covariates as per Table 4, NMBA therapy was not associated with 90-day mortality (adjusted HR 1.56 95% CI 0.55, 4.32,* P* = 0.392)

	Propensity score-matched cohort (*N* = 100)		
90-day mortality from ICU admission	Control (*N* = 50)	NMBA treatment (*N* = 50)	Total (*N* = 100)
Survived	38 (76%)	33 (66%)	71 (71%)
Died	12 (24%)	17 (34%)	29 (29%)
Total	50	50	100

**Table 8:** NMBA treatment (defined by > 3 days within 48 h from commencement of mechanical ventilation) vs controls on 90-day in-hospital mortality

	Propensity score-matched cohort (*N* = 324)		
90-day mortality from ICU admission	Control (*N* = 162)	NMBA treatment (*N* = 162)	Total (*N* = 362)
Survived	130 (80.3%)	81 (50.0%)	211 (65.1%)
Died	32 (19.8%)	81 (50.0%)	113 (34.9%)
Total	162	162	324

Prior to propensity score matching, neuromuscular blocking agent (NMBA) treatment, defined as more than 3 days of continuous use of NMBAs, within 48 h from commencement of invasive mechanical ventilation, was associated with 90-day mortality (unadjusted Cox regression; HR 2.57, 95% CI 2.02, 2.71, *p* < 0.001). After propensity score matching, 372 patients who received NMBA treatment were matched with 372 controls (using replacement). Using the propensity score-matched cohort and adjusting for covariates as per Table 4, NMBA therapy was associated with 90-day mortality (adjusted HR 1.73, 95% CI 1.27, 2.37, *p* = 0.001)

### Secondary outcome: ventilator-free days

In the PS-matched cohort at 28 days after commencement of IMV, VFD were 16 (IQR 0–25) in patients undergoing NMBA treatment and 25 (IQR 7–26) in the control group, and the sub-hazard ratio is 0.82 (95% CI 0.67, 1.00, *p* = 0.055). However, at 90 days after commencement of IMV, VFD were 77 (IQR 0–87) in patients undergoing NMBA treatment and 87 (IQR 0–88) in the control group; HR 0.86 (95% CI 0.69, 1.07; *p* = 0.177).

## Discussion

To the best of our knowledge, this is the largest, international observational study of patients with COVID-19 requiring mechanical ventilation to assess the impact of short-course NMBA treatment, commenced early during IMV, on 90-day in-hospital mortality. We found that NMBA use was common, specifically at European sites, and frequently applied in patients who presented hypercapnic and required higher levels of PEEP upon commencement of IMV. PS-matching analysis confirmed that early NMBA treatment did not result in lower mortality, while post hoc sensitivity analysis found increased mortality risk when NMBAs use was extended beyond 3 days. In addition, at 28 and 90 days, there were no between-group differences in days free of mechanical ventilation.

NMBAs are commonly used in critically ill patients who require IMV, but this practice has considerably changed throughout the years. In the 1980s, a survey from Great Britain reported NMBA administered in 90% of the patients on IMV [13], while in 2005 data from an international large cohort of mechanically ventilated patients reported NMBAs use merely in 13% of the patients [31]. The most recent clinical practice guidelines by the Society of Critical Care Medicine specified various indications for the use of NMBA in critically ill adult patients [32], among those patients with ARDS and PaO_2_/FiO_2_ < 150. In line with the original findings by Gainnier et al. demonstrating consistent oxygenation improvement in ARDS patients undergoing a 48-h course of NMBA [33], the confirmatory ACURASYS trial found that early administration of continuous cisatracurium for 48 h improved 90-day survival [15] and reduced risk of barotrauma. However, subsequent results from the Reevaluation of Systemic Early Neuromuscular Blockade (ROSE) trial failed to show reductions in mortality [16]. Meta-analyses emphasized potential limitations of those previous trials, i.e., heterogeneity in the use of sedation and prone positioning, selection bias, and crossover between treatment groups. Thus, to date, available evidence supports the use of NMBAs to reduce risks of barotrauma and to improve oxygenation [34–36], but without clear advantage in survival rate. ARDS caused by COVID-19 has broad similarities with historic ARDS caused by other etiologies [18, 37–39], although pulmonary blood flow derangement and resulting pulmonary shunt seem to play a primary role in COVID-19 ARDS [40]. As a result, various interventions previously employed for ARDS, such as prone position [41], ECMO [42], and NMBAs [20], were extensively applied in the COVID-19 population.

In the current study, we describe COVID-19 patients who required mechanical ventilation and who presented with moderate-to-severe hypoxemia. Various small case reports have demonstrated substantial ventilatory asynchrony in these patients [43–45] and adverse sequelae, such as pneumothorax or pneumomediastinum [46–48]. Patients receiving NMBA also frequently required adjunctive therapies for ARDS, suggesting that the severity of disease was a main driver in the use of these drugs. The COVID-19 population subset investigated in our analyses provides further value to our study; indeed, we identified patients with moderate-to-severe ARDS who received, early, a short course of NMBAs. This is in line with protocols applied in previous large randomized trials [15, 16]. Furthermore, in comparison with the ACURASYS and ROSE trials, we found that the COVID-19 population presented similar baseline impairment in respiratory function, and as in the ROSE trial, low tidal volume and high PEEP were applied and resulted in comparable airway plateau pressure. However, prone position was used much more frequently in the COVID-19 population and the improvements associated with its use [49, 50] might have offset any additional benefit related to NMBAs.

Courcelle and collaborators specifically investigated the effects of NMBAs in COVID-19 patients enrolled in French/Belgian ICUs. After PS matching, they did not find a significant difference in 28-day mortality [20]. Similar to these preliminary findings, our international, larger multicenter investigation also found no improvement in 90-day mortality in patients receiving a short course of NMBAs. Moreover, Courcelle found a median duration of NMBA use of 5 days; conversely, our study appraised only patients who received continuous NMBAs up to 3 days, and interestingly, we found higher hazard ratio of 90-day mortality when the use of NMBAs was extended more than 3 days. This finding provides further evidence on the risks associated with NMBAs in COVID-19 patients. Relative to Courcelles PS-matched cohort, our matched population was similar in age, gender proportion, and BMI and initial ventilatory settings. Yet, in the French/Belgian study, prone positioning was applied in over 90% and ECMO in 15% of the patients, in line with French expert consensus guidelines recommendations [51] and evidence in favor of the aforementioned interventions in COVID-19 patients [42, 50, 52]. Conversely, our dataset encompassed a global population of critically ill COVID-19 patients, including ICU centers both with and without access to modalities such as prone position and ECMO, which were applied in up to 69.6% and 16.7%, respectively. In the early waves of the pandemic, centers with the highest patient numbers acknowledged that treatments, such as prone positioning, could sometimes not be accomplished, due to staffing limitations and patient safety concerns [11]. Similarly, NMBA use at centers with overwhelming ICU surge may also be greater than in less resource-constrained environments.

Non-depolarizing NMBAs inhibit the acetylcholine (Ach) receptor on the motor endplate and are available as aminosteroid or benzylisoquinolinium compound, the latter being the first choice for ARDS patients [53]. Renal and hepatic disease can drastically prolong the clearance of aminosteroid NMBAs, and in these conditions benzylisoquinolinium agents are preferred, since they undergo spontaneous degradation via Hofmann elimination. The NMBA class used was not recorded in our dataset, but severe liver and chronic kidney diseases were present in only 2.9 and 5.7% of the patients treated with NMBAs, respectively. Of note, COVID-19 can cause a broad variety of neurological symptoms and sequelae [54] and has been associated with the development of anti-Ach receptors antibodies [55] and myasthenia gravis [56]. Hence, comprehensive research is needed in this field to elucidate whether underlying neurological mechanisms associated with COVID-19 lead to an increased risk of death when NMBA are administered, and caution should be applied.

## Strengths and limitations

To date, this is the first international report of a large group of mechanically ventilated COVID-19 patients with severe disease to investigate the effects of NMBA. Employing comprehensive PS matching analysis, we demonstrated no improvement in mortality with NMBA use. These findings were consistently corroborated in sensitivity analysis applying inverse probability of treatment weighting. In addition, we provided inferences not limited by clinical practice specific to single-country studies. Nonetheless, certain limitations must be highlighted. First, although we conducted a comprehensive comparative PS-matched analysis that resulted in significant homogeneity in the evaluated sub-cohorts, the observational nature of our study and risks of drawing causal inferences should be highlighted. While immortal time bias could be considered as a potential limitation of this study design, given the short duration between cohort entry and exposure to NMBA therapy, the magnitude of the bias is expected to be limited [57]. In addition, similar times from ICU admission to death were found in both groups. Yet, the risk of immortal time bias should also be considered for several interventions potentially associated with survival—such as prone positioning, ECMO, and corticosteroids—since only patients who survived long enough to receive these interventions were analyzed. Second, the analyzed dataset did not provide any information on the type of NMBA, doses, or adequacy of the block achieved; hence, we were unable to extrapolate the appropriateness of their clinical use. In addition, the time of commencement of NMBA treatment was not recorded; thus, although we included patients who received NMBAs up to 3 days, and we explored different treatment regimens in sensitivity analyses, potential discrepancy in the clinically indicated 2-day full course of NMBA treatment [15, 16] should be considered when interpreting the results. Third, a proportion of the studied population was admitted to the ICU early during the pandemic. Consequently, potential logistical limitations related to managing critically ill patients treated in newly developed and understaffed ICUs, due to shortages in ICU beds or healthcare providers, must be considered. Fourth, as shown in Fig. 4, residual discrepancy for Asia likely persisted post-matching. Further assessment on the potential reasons for this divergence suggested possible increased mortality in control patients from Asia. This could be related to the specific regional differences in ventilatory practice or application of adjunctive therapies, which should be further explored in future studies. Irrespective, potential random effect related to heterogenous practice among collaborating hospitals should be considered while interpreting our results. Fifth, the voluntary nature of site participation in the study must be emphasized, especially during a pandemic, as data may be skewed toward centers with enough resources to enter data. Lastly, as our observational study acquired data from routine clinical records, missing data could have biased our estimates.

## Conclusions

Our results derived by PS-matching analysis suggest that among patients with COVID-19 and moderate-to-severe ARDS, early administration of a short course of NMBAs did not result in improved 90-day in-hospital mortality. In addition, NMBA treatment did not impact ventilator-free days. Thus, the use of NMBAs should be cautiously assessed in this population, pending further studies that could elucidate specific indications for NMBAs in COVID-19.

## Supplementary Information

**Additional file 1**. Appendix.

## Data Availability

The datasets used and/or analyzed during the current study are available from the corresponding author on reasonable request.

## Abbreviations

ARDS: Acute respiratory distress syndrome
PaCO_2_: Arterial partial pressure of carbon dioxide
CI: Confidence interval
COVID-19: Coronavirus disease 2019
COVID-19–CCC/ECMOCARD: COVID-19 Critical Care Consortium incorporating the ExtraCorporeal Membrane Oxygenation for 2019 novel Coronavirus Acute Respiratory Disease
HR: Hazard ratio
ISARIC: International Severe Acute Respiratory and Emerging Infection Consortium
IMV: Invasive mechanical ventilation
IQR: Interquartile range
MAP: Mean arterial pressure
NMBAs: Neuromuscular blocking agents
PEEP: Positive end-expiratory pressure
PS: Propensity score
PaO_2_/FiO_2_: Ratio between arterial partial pressure of oxygen and inspiratory fraction of oxygen
SD: Standard deviation
SARS-CoV-2: Severe acute respiratory syndrome coronavirus 2
SPRINT-SARI: Short PeRiod IncideNce sTudy of Severe Acute Respiratory Infection
